# NCME-Net: Nuclear cataract mask encoder network for intelligent grading using self-supervised learning from anterior segment photographs

**DOI:** 10.1016/j.heliyon.2024.e34726

**Published:** 2024-07-17

**Authors:** Jiani Zhao, Cheng Wan, Jiajun Li, Zhe Zhang, Weihua Yang, Keran Li

**Affiliations:** a College of Electronic and Information Engineering /College of Integrated Circuits, Nanjing University of Aeronautics and Astronautics, Nanjing, Jiangsu, 211106, China; b Eye Hospital, Nanjing Medical University, Nanjing, Jiangsu, 210029, China; c Shenzhen Eye Institute, Shenzhen Eye Hospital, Jinan University, Shenzhen, Guangdong, 518040, China

**Keywords:** Cataract, Deep learning, Self-supervision, Hybrid model

## Abstract

Cataracts are a leading cause of blindness worldwide, making accurate diagnosis and effective surgical planning critical. However, grading the severity of the lens nucleus is challenging because deep learning (DL) models pretrained using ImageNet perform poorly when applied directly to medical data due to the limited availability of labeled medical images and high interclass similarity. Self-supervised pretraining offers a solution by circumventing the need for cost-intensive data annotations and bridging domain disparities. In this study, to address the challenges of intelligent grading, we proposed a hybrid model called nuclear cataract mask encoder network (NCME-Net), which utilizes self-supervised pretraining for the four-class analysis of nuclear cataract severity. A total of 792 images of nuclear cataracts were categorized into the training set (533 images), the validation set (139 images), and the test set (100 images). NCME-Net achieved a diagnostic accuracy of 91.0 % on the test set, a 5.0 % improvement over the best-performing DL model (ResNet50). Experimental results demonstrate NCME-Net's ability to distinguish between cataract severities, particularly in scenarios with limited samples, making it a valuable tool for intelligently diagnosing cataracts. In addition, the effect of different self-supervised tasks on the model's ability to capture the intrinsic structure of the data was studied. Findings indicate that image restoration tasks significantly enhance semantic information extraction.

## Highlights

• We proposed a simplified grading method for nuclear cataracts based on AREDS No. 4.
• The NCME-Net achieved 91% accuracy in diagnosing nuclear cataracts using self-supervised learning.
• This study contributes to the transformation of digital slit lamp imaging into an intelligent diagnosis.

## 1 Introduction

Cataract refers to the clouding of the lens caused by protein denaturation [1] and accounts for 1 million cases of blindness annually, making them the leading cause of blindness Worldwide [[2], [3], [4]]. Recognized by the World Health Organization as a preventable cause of visual disability, cataracts are usually treated surgically [5]. However, due to low public awareness and socioeconomic constraints, annually, approximately 4 million patients with operable cataracts delay seeking treatment until they lose vision in one eye [6,7]. Moreover, the healthcare system cannot provide prompt services to patients during unanticipated events, such as coronavirus disease 2019 (COVID-19). During the COVID-19 pandemic, compared with 2019, the number of cataract surgeries in 2020 decreased by 97 % and 95 % in the United States and India, respectively [8,9]. The failure to expand ophthalmic medical care has resulted in a substantial healthcare gap. Blindness association programs and free clinics managed by non-governmental organizations can mitigate some of the burden on the healthcare system. However, patients with undiagnosed cataracts continue to pose a challenge [[10], [11], [12]].

Automated high-precision cataract grading is a critical need, and the COVID-19 pandemic has further underscored its importance [8,9]. Clinically, ophthalmologists use slit lamps to capture photographs of the anterior segment of the eye, focusing on the nucleus region to grade cataracts based on opacity. This manual examination process is time-consuming and subjective, highlighting the need for automated grading systems. Hasan proposed an ensemble machine learning model using CLAHE-enhanced retinal fundus images for cataract detection with high precision [13]. Anwer et al. found that logistic regression achieved the highest accuracy in classifying retinal fundus images from the ODIR dataset [14]. Qian et al. achieved a diagnostic accuracy of 96.0 % in cataract classification by using SqueezeNet with transfer learning [15]. Zhou et al. proposed a discrete state transition neural network based on prior knowledge, demonstrating enhanced applicability in complex medical imaging [16]. Zhang et al. proposed RCRNet to optimize cataract recognition efficiency and performance by using clinical priors [17]. Gu proposed Ranking-MFCNet for fine-grained cataract severity classification based on AS-OCT images by utilizing multiscale feature calibration and external attention augmentation [18]. Furthermore, Zhang enhanced cataract grading in AS-OCT images by employing region-based integration and recalibration attention mechanisms [19], and Xu et al. developed GLA-Net by integrating global-local attention to enhance hierarchical representation learning [20]. Song et al. combined multiple binary classifiers for semi-supervised cataract classification, achieving an accuracy of 88.6 % [21].

Deep learning (DL) techniques enable intelligent cataract grading by avoiding the limitations of traditional detection methods. However, applying DL models to medical images poses significant challenges. First, these models require substantial data for effective diagnosis. Second, the characteristics of the weights in ImageNet differ significantly from those needed for medical image classification. Consequently, it is challenging to apply DL models with ImageNet pretrained weights directly to medical data, which is often characterized by small sample sizes and high interclass similarities [22]. Self-supervised learning strategies can eliminate the need for cost-intensive data labeling and bridge knowledge gaps. This concept has been applied to medical image processing by using a transformer model [23]. Although transformer models outperform convolutional neural networks (CNNs) by analyzing all image data, they may not perform well on limited datasets [24].

**Fig. 1:**
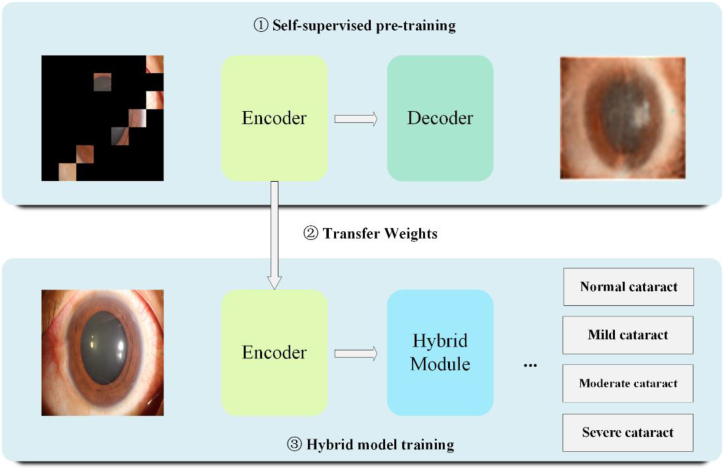
Structure of NCME-Net.

To address these challenges, in this paper, we proposed the nuclear cataract mask encoder network (NCME-Net), which combines the strengths of CNNs and transformers and utilizes self-supervised pretraining. NCME-Net can handle limited sample sizes and high interclass similarities typical of medical image datasets by leveraging the CNN's ability to extract localized features and the transformer's ability to capture global context. The proposed model utilizes self-supervised pretraining to learn to reconstruct masked portions of the images, thereby gaining a deeper understanding of the underlying data structure without requiring labeled data. This approach helps the model adapt more effectively to the medical image domain, thus yielding improved performance in cataract grading tasks. The structure of the proposed model is illustrated in Fig. 1. By employing this hybrid approach, NCME-Net provides more accurate and objective grading of nuclear cataracts, reducing reliance on manual assessment and enhancing diagnostic efficiency in clinical settings.

## 2 Methods

### 2.1 Data acquisition

**Fig. 2:**
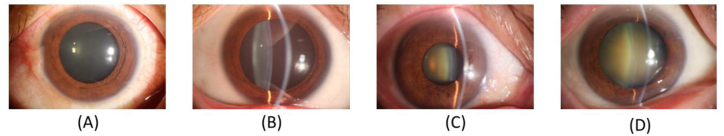
The anterior-segment photographs of each category: (A) Normal; (B) Mild cataract; (C) Moderate cataract; (D) Severe cataract.

The slit lamp is a non-invasive lamp that is used to examine both superficial and deep eye lesions, aiding in determining cataract severity. Based on slit-lamp examination results, doctors assess cataract severity using various grading systems such as LOCSIII, Wisconsin, and AREDS No. 4 guidelines [[25], [26], [27]]. However, these grading systems are complex and require experienced ophthalmologists, leading to some degree of subjectivity in the diagnosis [28,29]. To avoid subjectivity in manual assessment, in this study, we recategorized nuclear cataracts based on the AREDS No. 4 guidelines for a more objective determination of nuclear cataract severity. Specifically, criterion 1 was defined as normal, criteria 2 and 3 as mild, criteria 4 and 5 as moderate, and criteria 6 and 7 as severe cataracts, resulting in four distinct categories (Fig. 2). This simplified classification provides clearer and more consistent determination criteria for intelligent cataract grading.

All participants provided informed consent, and the study design was approved by the review board of the Medical Ethics Committee of Shenzhen Eye Hospital. Following the Declaration of Helsinki, all patient-related information was anonymized during data acquisition. Data were obtained from the Shenzhen Eye Hospital and the Eye Hospital of Nanjing Medical University and then clinically graded by ophthalmologists. Three ophthalmic experts conducted a diagnostic assessment of slit-lamp photographs (Sunkingdom LS-7DE Slitlamp, Sunkingdom, Chongqing, China) by using a double-blind methodology. In cases of diagnostic discordance between two physicians, a third senior ophthalmologist provided the final adjudication to ascertain the definitive rating level for the respective photograph.

For model training and evaluation, 792 anterior-segment photographs were used. To ensure fairness, random number seeds were used to divide the dataset into training, validation, and test sets. Specifically, 100 slit-lamp photos were designated as the test set, and the remaining data were split into training and validation sets in an 8:2 ratio. The proportion of cataract samples with various severity levels was similar across all three datasets. Table 1 presents the details of the data division. No data augmentation methods, such as image inversion, were employed to verify that NCME-Net can overcome high similarity between data classes in small datasets.

**Table 1:** Number of anterior-segment photographs in the training, validation, and test sets from the clinical classification of cataract.

Simplified Classification of AREDS No. 4 Guidelines	No. of Anterior Segment Photographs (% Total)		
	Training sets	Validation sets	Test sets
No-cataract	87 (15.7)	22 (15.8)	16 (16.0)
Mild cataract	152 (27.5)	38 (27.3)	28 (28.0)
Moderate cataract	224 (40.5)	56 (40.3)	40 (40.0)
Severe cataract	90 (16.3)	23 (16.6)	16 (16.0)
Total	553(100.0)	139 (100.0)	100 (100.0)

### 2.2 NCME-Net structure

NCME-Net, a hybrid model based on self-supervised pretraining strategies, addresses the challenges typically encountered in medical image processing. CNNs excel at local feature extraction, whereas transformers capture global contextual information. By combining these two approaches, NCME-Net effectively captures the relationships between various locations in the feature map, thereby enhancing the focus on essential features. The architecture of NCME-Net comprises three fundamental elements: self-supervised pretraining, weight transfer, and hybrid model training. Details regarding the structure of NCME-Net are presented in Table 2.

**Table 2:** Architecture of NCME-Net for the analysis of nuclear cataract severity by using anterior-segment images.

layer name	NCME-Net	output size
conv1	7x7, 64, stride 2	112x112
Conv2_x	3x3 max pool, stride 2	56x56
	$\left[\begin{array}{cc} 1\times 1\text{,} & 64\\ 3\times 3\text{,} & 64\\ 1\times 1\text{,} & 256 \end{array}\right]\times 3$	
Conv3_x	$\left[\begin{array}{cc} 1\times 1\text{,} & 128\\ 3\times 3\text{,} & 128\\ 1\times 1\text{,} & 512 \end{array}\right]\times 3$	28x28
Conv4_x	$\left[\begin{array}{cc} 1\times 1\text{,} & 256\\ 3\times 3\text{,} & 256\\ 1\times 1\text{,} & 1024 \end{array}\right]\times 3$	14x14
Conv5_x	$\left[\begin{array}{cc} 1\times 1\text{,} & 512\\ 3\times 3\text{,} & 512\\ 1\times 1\text{,} & 2048 \end{array}\right]\times 3$	7x7
DPCT	3x3, 2048, stride 2	1x1

**Fig. 3:**
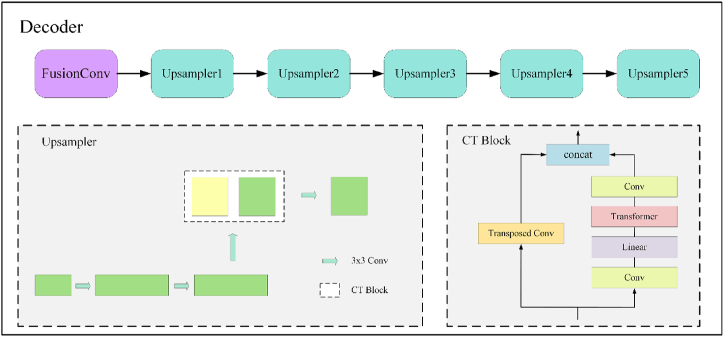
Structure of the decoder.

**Self-supervised pretraining:** Through self-supervised pretraining, NCME-Net addresses inconsistencies in DL models trained on natural images when applied to medical data, thereby enhancing classification performance. This pretraining utilizes an encoder–decoder architecture with ResNet50, the best-performing model on validation and test sets, as the encoder. The decoder employs a hybrid upsampling technique combining CNN and transformer components, referred to as the CT block, as illustrated in Fig. 3.

The FusionConv module, comprising two convolutional layers, adjusts the number of channels and integrates the outputs, ensuring a smooth transition between the encoder and decoder. The CT block's hybrid upsampling aligns NCME-Net better with the data feature distributions of the hybrid model, thereby enhancing classification performance. To achieve this, the CT block changes the number of channels by using a convolutional layer, expands the input features fourfold with a linear layer, and merges the results from the CNN and transformer components along the channel dimension to complete the upsampling process. The structure of the transformer block is shown in Fig. 5 (right). To increase the complexity of the self-supervised pretraining task, a no-skip connection strategy is used, transferring features from the encoder to the decoder without skip connection.

**Fig. 4:**
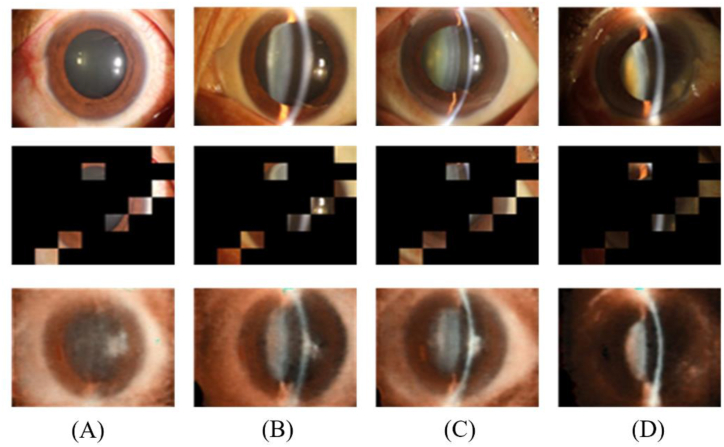
Image restoration results: (A) Normal; (B) Mild cataract; (C) Moderate cataract; (D) Severe cataract.

Self-supervised pretraining allows the model to learn the underlying structure and feature representation of the data through an image restoration task, which involves masking the input image and revealing only a portion of it. The encoder–decoder architecture then restores the image, improving the model's performance in downstream tasks. The self-supervised tasks were trained and validated only on the training and validation sets, with the test set not being involved to ensure objectivity and fairness. The image restoration results for normal, mild, moderate, and severe cataracts at a mask ratio of 1/7 are shown in Fig. 4.

**Fig. 5:**
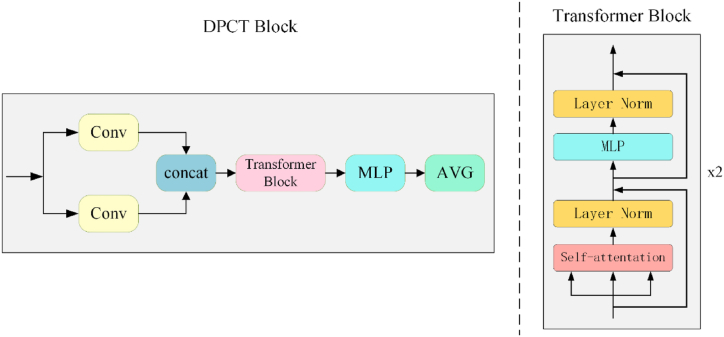
Structure of the DPCT block.

**Weight transfer:** After completing self-supervised pretraining, the encoder and its weights are transferred to serve as the backbone network of the hybrid model. The weights from pretraining are used as the initialization weights for the backbone network. This weight transfer enables NCME-Net to utilize the knowledge acquired during pretraining to extract advanced image features, enhancing performance in downstream tasks.

**Hybrid model training:** The hybrid model comprises a backbone network and a Dual Path ConvTransformer (DPCT) block, as shown in Fig. 5 (left). The DPCT block begins with two 1 × 1 convolutional layers to halve the number of channels in the image. It then splices the feature image output from the two convolution layers in the channel dimensions to restore the original feature map size. This operation allows the DPCT block to integrate features from different channels, forming a more advanced feature representation.

To address the limitation of the encoder processing data only locally, we introduced the long-term dependence of the transformer module to capture data features. Originally applied in machine translation, the transformer model can process global data information [30]. The improved performance of the transformer model has led to its increasing application in image processing [23,31]. Through the self-attention mechanism for modeling global dependency relationships, the transformer model provided a novel solution for computer vision tasks and improved the model performance. The self-attention mechanism can be expressed as follows:

(1) $$Attentation\left(Q,K,V\right)=softmax\left(\frac{QK^{T}}{\sqrt{d_{k}}}\right)V\text{.}$$

in the DPCT block, two layers of transformer blocks are used. The structure of the transformer block is illustrated in Fig. 5 (right). The transformer mechanism enables NCME-Net to capture relationships between global and local features within the feature graph, thereby improving classification performance. Finally, the MLP layer transforms the output channels of the transformer block to four, representing the probabilities of normal, mild, moderate, and severe cataracts.

### 2.3 Self-supervised pretraining tasks

**Fig. 6:**
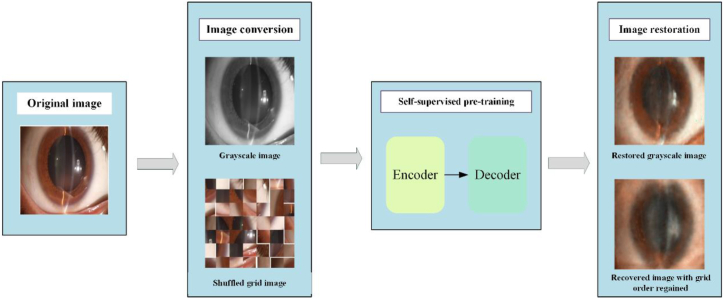
Other self-supervised pretraining tasks.

Various self-supervised pretraining tasks can enhance a model's ability to extract semantic information by improving its understanding of intrinsic data structures. We tested two typical tasks, namely grayscale image coloring and shuffled grid image reconstruction, to investigate their effects on the feature-capturing ability and downstream performance of the encoder. The grayscale image coloring task helped the model learn the global shape and scene information of the image by predicting color attributes. In contrast, the shuffled grid image reconstruction task improved the model's understanding of local details by destroying and reconstructing spatial information. Using the mixed upsampling encoder–decoder structure of the CNN and transformer, the model effectively recovered colors for the relatively simple grayscale image coloring task but was less effective for the more complex raster order disruption reconstruction task (Fig. 6).

### 2.4 Statistical method

In this study, nuclear cataracts were classified into four categories: normal, mild, moderate, and severe. The model's performance was evaluated against the grading results provided by ophthalmologists, which served as the gold standard. Both binary and multi-classification metrics were used for assessment. For the binary metrics, the diagnostic results of NCME-Net were divided into four subtasks, and the average of the binary indicators across these subtasks was used for the final evaluation. These binary indicators included the positive predictive value (PPV), negative predictive value (NPV), sensitivity (SE), and specificity (SP), whose formulas are as follows:

(2) $$\text{PPV}=\frac{TP}{TP+FP}\text{,}$$

(3) $$\text{NPV}=\frac{TN}{TN+FN}\text{,}$$

(4) $$\text{SE}=\frac{TP}{TP+FN}\text{,}$$

(5) $$\text{SP}=\frac{TN}{TN+FP}\text{,}$$

where TP, TN, FP, and FN represent true positives, true negatives, false positives, and false negatives, respectively. Receiver operating characteristic (ROC) curves were plotted, and the area under the curves (AUCs) was calculated to evaluate the performance of the model across various thresholds. An AUC greater than 0.85 indicated excellent grading performance.

To compare the model's results with ophthalmologists' assessments, we used the kappa value to assess the agreement between the model's predictions and the ophthalmologists' grades, with higher kappa values indicating better agreement. The kappa value was calculated as follows:

(6) $$\text{kappa}=\frac{p_{o}-p_{e}}{1-p_{e}}\text{,}$$

(7) $$p_{e}=\frac{a_{1}\times b_{1}+a_{1}\times b_{1}+\ldots +a_{c}\times b_{c}}{n\times n}\text{,}$$

where $p_{0}$ is the overall classification accuracy, $a_{i}$ denotes the actual number of Type I samples, and $b_{i}$ is the number of samples predicted by class I.

### 2.5 Implementation

NCME-Net was developed using Python 3.7.11 with PyTorch and trained on an NVidia 3090Ti GPU (24 GB). The Adam optimizer and cosine annealing algorithm were used to adjust the learning rate during model training. The training dataset was augmented using HSV color space changes, random adjustments to brightness and contrast, Gaussian blur, and grid distortion, with probabilities of 0.1, 0.1, 0.3, and 0.3, respectively, to increase diversity and prevent overfitting. A cross-entropy loss function with label smoothing was used to evaluate the gap between predicted and actual labels and improve the robustness of the model [31].

## 3 Results

### 3.1 Results of dataset

DL models showed varying adaptability to the same data. The experimental outcomes of testing the two types of models (CNN and transformer) for classifying the four types of nuclear cataracts are presented in Table 3. In the validation set, EfficientNetV2 and ResNet50 achieved an accuracy of 85.5 %, but ResNet50 demonstrated superior generalization with a diagnostic accuracy of 86.0 % in the test sets. Although effective with large-scale data, the transformer model did not perform as well with a smaller sample size. Considering these results, ResNet50 was selected as the encoder.

**Table 3:** Model performance.

Model		Accuracy (%)	
		Validation sets	Test sets
CNN	EfficientNetV2 [32]	**85.5**	85.0
	NASNet [33]	83.3	82.0
	RegNet [34]	84.8	83.0
	ResNeXt [35]	83.3	85.0
	ResNet50 [36]	**85.5**	**86.0**
Transformer	ViT [24]	83.3	83.0
	Swin [37]	84.1	85.0

### 3.2 NCME-Net performance evaluation across various mask ratios

**Fig. 7:**
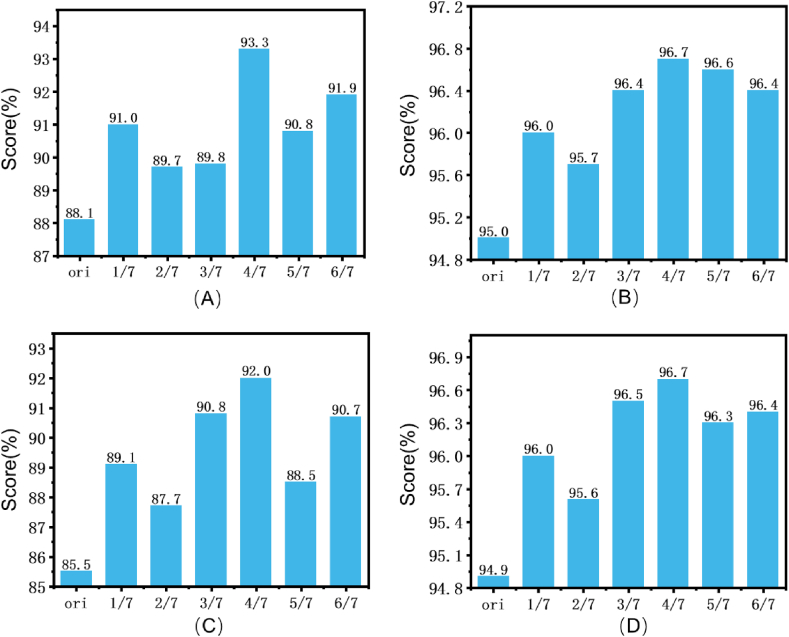
Results of the assessment of dichotomous indicators: (A) PPV; (B) NPV; (C) SE; (D) SP. Ori stands for ResNet50. 1/7 to 6/7 represent the performance of NCME-Net under different shielding rates.

After transferring the self-supervised pretraining weights, the performance of NCME-Net improved based on the optimal ResNet50 model. Binary indicators, namely PPV, NPV, SE, and SP, were used to evaluate ResNet50 and NCME-Net under different mask ratios (Fig. 7). The best performance for NCME-Net was achieved with a mask ratio of four-sevenths, resulting in PPV, NPV, SE, and SP values of 93.3 %, 96.7 %, 92.0 %, and 96.7 %, respectively. The SE of NCME-Net was 6.5 % higher than that of ResNet50, showing a significant improvement in identifying positive cases.

**Fig. 8:**
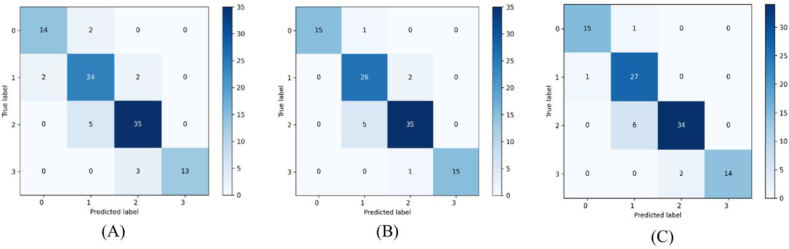
Confusion matrices: (A) ResNet50; (B) 4/7; (C) 6/7. 0 for normal, 1 for mild cataracts, 2 for moderate cataracts, and 3 for severe cataracts.

Confusion matrices revealed the diagnostic results for ResNet50 and NCME-Net in grading nuclear cataracts. With mask ratios of four-sevenths and six-sevenths, NCME-Net achieved a diagnostic accuracy of 91.0 % across all four nuclear cataract categories (Fig. 8). Misclassifications occurred only between adjacent classes, highlighting the high interclass similarity in cataracts. Self-supervised pretraining helped mitigate these diagnostic challenges stemming from high interclass similarity. At a mask ratio of four-sevenths, NCME-Net accurately distinguished between normal and mild cataracts, avoiding misclassification of mild cataracts as normal. Moreover, at a mask ratio of six-sevenths, no mild cataracts were misdiagnosed as moderate. Furthermore, compared with ResNet50, NCME-Net reduced the misdiagnosis of severe cataracts by two and one cases at mask ratios of four-sevenths and six-sevenths, respectively, thus minimizing the misclassification of severe cataracts. The ability of NCME-Net to effectively discriminate between normal and severe cataracts, despite the availability of limited data, demonstrates its ability to overcome data similarity in small datasets.

**Fig. 9:**
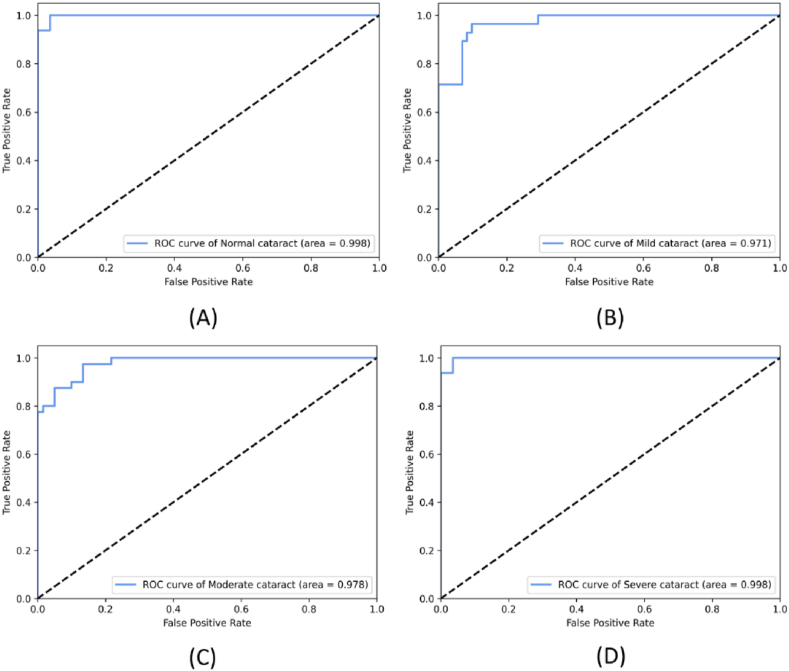
Receiver operating characteristic curves: (A) Normal; (B) Mild cataract; (C) Moderate cataract; (D) Severe cataract.

The ROC curve demonstrates the model's performance at different thresholds. The AUC for normal, mild, moderate, and severe cataracts were 99.8 %, 97.1 %, 97.8 %, and 99.8 %, respectively, with an average AUC of 98.6 % (Fig. 9). These results demonstrate NCME-Net's high diagnostic value for intelligent cataract grading.

### 3.3 Performance of different self-supervised pretraining tasks

The effect of the two proposed self-supervised pretraining tasks, namely grayscale image colorization and grid shuffling reconstruction, was studied. After self-supervised pretraining, the encoder and its weights were transferred, and fine-tuning experiments were conducted with NCME-Net. As can be observed from Table 4, pretraining tasks enhanced the model's classification capability compared to training from scratch. The shuffled-grid image reconstruction task slightly outperformed image coloring, but both tasks provided limited improvements compared to image restoration. This demonstrates that image restoration is a more effective self-supervised pretraining approach, significantly boosting model performance in downstream tasks by capturing the inherent structure of images.

**Table 4:** Results of cataract severity quadric classification with transfer learning from different self-supervised pretraining tasks.

self-supervised pretraining tasks	Accuracy(%)	Kappa(%)
restored grayscale image	88.0	83.0
shuffled grid image reconstruction	89.0	84.5
image restoration(NCME-Net 4/7)	91.0	87.3
no self-supervised pretraining	87.0	81.6

## 4 Discussion

China has the highest number of blind individuals worldwide, with cataracts accounting for nearly 50 % of these cases [38,39]. Many countries face a shortage of clinicians to meet patients' needs, resulting in the gradual deterioration of patients' vision and severely affecting their daily lives [40]. The burden of treatable blindness caused by cataracts increases as the population ages. Furthermore, the grading of nuclear cataracts heavily relies on ophthalmologists’ subjective judgment, leading to variability in diagnoses and time-consuming procedures. Achieving efficient cataract grading remains a challenge for healthcare systems. Therefore, applying DL methods to evaluate cataract severity across the four categories is essential.

In this study, we reclassified the seven cataract nuclear grading guidelines proposed in AREDS No. 4 into four categories (normal, mild, moderate, and severe) by using anterior-segment photographs. This simplified approach reduces subjectivity in grading by physicians, thereby improving accuracy in assessing cataract severity. Moreover, leveraging self-supervised pretraining, we developed a decoder with mixed upsampling by using CNN and transformer architectures to explore structural and feature representations for image restoration. Deep mining of image information through image restoration learning adapted the initial weights of the model to the data, avoiding problems such as weight mismatch caused by the direct transfer of the ImageNet weights and enhancing the model performance. In addition, we proposed a hybrid module that integrates encoder features from both architectures to overcome the limitations of CNNs in extracting localized information. Experimental results demonstrate that NCME-Net improved diagnostic accuracy across six different mask ratios, eliminating randomness in Dl grading and overcoming the challenges posed by high interclass similarity when dealing with a limited number of samples.

Although self-supervised pretraining has shown success in small datasets, its application in medical image analysis has received limited attention. Zhou et al. demonstrated that networks with prior information are better suited for grading complex images [14]. RCRNet utilizes clinical priors of asymmetric opacity to enhance efficiency and performance in cataract recognition [17]. Gu's Ranking-MFCNet incorporates multiscale feature calibration and external attention augmentation for fine-grained severity classification on AS-OCT images [18]. These methods emphasize unique architectural designs or attention mechanisms tailored to address the challenges encountered in cataract grading. In this study, we proposed self-supervised pretraining for four-class severity grading of nuclear cataracts. As opposed to other methods, NCME-Net utilizes self-supervised pretraining to effectively mine cataract data and initialize weights suitable for experimental conditions, thus minimizing the need for labeled data and manual preprocessing and avoiding subjective adjustments such as manual distribution changes or lesion region cropping [13,15]. Unlike approaches that emphasize denoising before grading, NCME-Net simplifies the workflow by reducing preprocessing requirements and enhancing the model's adaptability to the varying image conditions encountered in clinical settings [41,42].

NCME-Net effectively distinguished between normal, mild, and severe cataracts, demonstrating its effectiveness in diagnosing cataract severity. However, this study had some limitations. First, compared to the original model, NCME-Net did not address the problem of moderate cataracts being misdiagnosed as mild cataracts by existing models. Second, in the study, NCME-Net was evaluated without self-supervised pretraining, focusing on overall accuracy and kappa value assessment.

As can be observed i n Table 4, NCME-Net achieved 1 % higher accuracy than ResNet50 without self-supervised pretraining. Despite its strong diagnostic capabilities, NCME-Net faces challenges in achieving more accurate classifications without self-supervised pretraining, as indicated by the experimental results. Moreover, self-supervised pretraining enhances the model's ability to learn data characteristics, thus improving its performance. However, this process is time-consuming; for example, training the image restoration task for six different mask ratios on an NVidia 3090Ti GPU (24 GB) took 2.5 days, which could pose challenges for studies with limited equipment. Although NCME-Net performs well in recognizing cataract severity, it requires self-supervised pretraining to improve feature extraction capability for accurate grading results. Furthermore, the cost of self-supervised pretraining must be further optimized.

Although NCME-Net achieved good performance in this experimental dataset, implementing NCME-Net in a real clinical setting presents significant challenges and considerations. Regular updates and retraining with new data are essential to adapt NCME-Net to evolving patient needs. Continuous monitoring and validation of NCME-Net's performance in clinical settings are crucial for ensuring long-term reliability. Moreover, the self-supervised pretraining process is time-consuming and requires substantial computational resources. As such, optimizing this process to reduce training time and resource requirements is essential. In future research, we will focus on three aspects of cataract nucleus grading: (1) developing a simpler and more efficient task to reduce self-supervised pretraining time, (2) expanding the dataset to leverage self-supervised pretraining for extracting universally applicable initial weights suitable for cataract grading, and (3) exploring the integration of prior knowledge to enhance the model's sensitivity to regions of interest (ROIs) and improve cataract grading accuracy.

## 5 Conclusion

Cataracts significantly contribute to global visual impairment, necessitating timely intervention to preserve vision. In this paper, we proposed NCME-Net, a hybrid model that utilizes self-supervised learning to diagnose cataract severity with an impressive accuracy of 91 %. This intelligent diagnostic technology, leveraging anterior-segment photographs and DL algorithms, holds promise for providing a targeted intelligent diagnosis in medically underserved rural areas and supporting cataract screening in primary healthcare settings. Early detection and intervention through NCME-Net can ultimately improve the quality of vision and enhance the quality of life of affected individuals.

## Ethics approval

This study was reviewed and approved by the Medical Ethics Committee of Shenzhen Eye Hospital with the approval number: 2024KYPJ013, dated February 6, 2024.

## Financial Support

This research did not receive any specific grant from funding agencies in the public, commercial, or not-for-profit sectors.

## Data availability statement

The raw data supporting the conclusions of this article will be made available by the authors, without undue reservation.

## CRediT authorship contribution statement

**Jiani Zhao:** Writing – original draft, Validation, Software, Methodology. **Cheng Wan:** Writing – original draft, Validation, Methodology. **Jiajun Li:** Methodology, Investigation, Data curation. **Zhe Zhang:** Methodology, Investigation, Data curation. **Weihua Yang:** Writing – review & editing, Methodology, Investigation, Data curation, Conceptualization. **Keran Li:** Writing – review & editing, Investigation, Data curation, Conceptualization.

## Declaration of competing interest

The authors declare that they have no known competing financial interests or personal relationships that could have appeared to influence the work reported in this paper.
